# Recurrent Losses and Rapid Evolution of the Condensin II Complex in Insects

**DOI:** 10.1093/molbev/msz140

**Published:** 2019-07-04

**Authors:** Thomas D King, Christopher J Leonard, Jacob C Cooper, Son Nguyen, Eric F Joyce, Nitin Phadnis

**Affiliations:** 1 School of Biological Sciences, University of Utah, Salt Lake City, UT; 2 Department of Genetics, Penn Epigenetics Institute, Perelman School of Medicine, University of Pennsylvania, Philadelphia, PA

**Keywords:** condensin, chromosome pairing, molecular evolution

## Abstract

Condensins play a crucial role in the organization of genetic material by compacting and disentangling chromosomes. Based on studies in a few model organisms, the condensins I and II complexes are considered to have distinct functions, with the condensin II complex playing a role in meiosis and somatic pairing of homologous chromosomes in *Drosophila*. Intriguingly, the *Cap-G2* subunit of condensin II is absent in *Drosophila melanogaster*, and this loss may be related to the high levels of chromosome pairing seen in flies. Here, we find that all three non-SMC subunits of condensin II (*Cap-G2*, *Cap-D3*, and *Cap-H2*) have been repeatedly and independently lost in taxa representing multiple insect orders, with some taxa lacking all three. We also find that all non-Dipteran insects display near-uniform low-pairing levels regardless of their condensin II complex composition, suggesting that some key aspects of genome organization are robust to condensin II subunit losses. Finally, we observe consistent signatures of positive selection in condensin subunits across flies and mammals. These findings suggest that these ancient complexes are far more evolutionarily labile than previously suspected, and are at the crossroads of several forms of genomic conflicts. Our results raise fundamental questions about the specific functions of the two condensin complexes in taxa that have experienced subunit losses, and open the door to further investigations to elucidate the diversity of molecular mechanisms that underlie genome organization across various life forms.

## Introduction

The molecular machinery involved in the fundamental processes of genome organization is essential for all organisms and is deeply conserved. Condensins are key players in the tasks of compacting and disentangling chromosomes to ensure proper segregation of genetic material (Hirano and Mitchison 1994; Hirano 2005). All cellular life on Earth, including bacteria, archaea, fungi, plants, and animals, possess condensins or an analogous protein complex (Losada and Hirano 2005; Hirano 2016). The function of condensins is so integral to chromosome mechanics that they are thought to have arisen before histones (Kalitsis et al. 2017).

Two distinct condensin complexes are present in most multicellular eukaryotes. Condensins I and II are pentameric complexes that share a hinge structure made up of the *SMC2* and *SMC4* subunits. The non-SMC subunits of condensin I consist of *Cap-H*, a kleisin that serves a scaffold and linker, and the HEAT-repeat subunits *Cap-D2* and *Cap-G*, which bind *Cap-H2*. Analogously, in the condensin II complex, the kleisin *Cap-H2* subunit is bound by the *Cap-D3* and *Cap-G2* subunits (Hirano 2016). The functions of these eukaryotic condensin complexes are complementary: condensin I compresses chromosomes laterally and condensin II compresses them axially (Shintomi and Hirano 2011; Bauer et al. 2012; Green et al. 2012). Although both complexes are critical for chromosome segregation during mitosis, in interphase, condensin I is enriched in the cytoplasm whereas condensin II predominates in the nucleus (Ono et al. 2003; Gerlich et al. 2006; Hirano 2016).

Condensins use ATPase activity to fuel the asymmetric extrusion of DNA loops, allowing them to disentangle chromosomes, separate homologs, and compact chromatin (Goloborodko et al. 2016; Ganji et al. 2018). In addition to its role in cell division, condensin II contributes to the structure and organization of interphase chromosomes. Although the condensin II complex has several varied functions ranging from promoting polytene disassembly to regulating nuclear organization, its overall role is as a master regulator of chromosome individualization, consistent with its ability to untangle and separate neighboring chromatin fibers or chromosome territories (Goloborodko et al. 2016; Ganji et al. 2018; Rosin et al. 2018). In mice and flies, condensin II antagonizes clustering of pericentric heterochromatin (Bauer et al. 2012; Joyce et al. 2012; Nishide and Hirano 2014). Studies in *Drosophila* have also shown that the condensin II complex antagonizes the interhomolog pairing of chromosomes in somatic cells (Hartl, Smith, et al. 2008; Hartl, Sweeney, et al. 2008), and genome-wide screens have confirmed it as a central player in controlling homolog pairing behavior (Bateman and Wu 2008; Bateman et al. 2012; Joyce et al. 2012).

Intriguingly, the *Cap-G2* subunit of condensin II is absent in *Drosophila melanogaster* (Herzog et al. 2013). This loss in *Drosophila* is surprising, given the conservation of condensin II across most eukaryotes and its central role in essential cellular processes. *Drosophila* appear exceptional not only in their lack of *Cap-G2*, but also with regards to their nuclear organization. Flies align all pairs of homologs, end-to-end, in essentially all somatic tissues, a dramatic phenomenon not observed in any other clade (McKee 2004). When pairing is seen in non-Dipteran species, it is often localized, transient, and associated with unusual or diseased states such as tumor cells in humans (Scherthan et al. 1994; Riesselmann and Haaf 1999; Xu et al. 2006; Koeman et al. 2008). In *Drosophila*, pairing of homologous chromosomes in somatic cells is critical for phenomena such as transvection, in which alleles and/or regulatory elements interact and act upon each other interchromosomally (Lewis 1954; Wu and Morris 1999; Duncan 2002).

Given the importance of the condensin II complex in regulating pairing, we conjectured that the seemingly anomalous prevalence of pairing and the equally puzzling lack of a condensin II subunit in flies could be linked. However, it remains unclear when and how Dipterans evolved the drastic change in global nuclear organization enabling somatic homolog pairing, and whether this widespread pairing evolved independently or coincident with the loss of *Cap-G2*. Here, we investigate patterns of condensin II evolution, and the implications of these patterns for chromosome pairing in *Drosophila* and other insect species. We discovered that Dipterans are not unique in having lost a condensin II subunit. Instead, components of the complex have been repeatedly and independently lost in multiple insect lineages, with some taxa missing the condensin II-specific subunits altogether—a phenomenon not previously reported in multicellular eukaryotes. To explore the impact of these subunit losses on nuclear organization, we took advantage of the robust homologous chromosome pairing in *Drosophila* as readout of interphase condensin II activity. We developed Oligopaint DNA-FISH probes (Beliveau et al. 2012) and quantified pairing frequencies across several insect orders with differing complements of condensin II subunits. Surprisingly, our results show that condensin II subunit losses have no relationship with pairing prevalence, and no other taxa display somatic homologous pairing to the extent seen in Dipterans. This finding suggests that factors other than condensin II complex composition have important roles in the regulation of pairing and perhaps interphase chromosome compaction in general across insects. Finally, we show that both condensin complexes and the related cohesin complex have evolved rapidly under recurrent positive selection across multiple taxa, including *Drosophila* and several mammal clades, which suggest their participation in an evolutionary arms race driven by genetic conflict. Together, our study paints a dynamic and counterintuitive view of the function and evolutionary history of condensins, and opens the door to comparative functional studies of genome organization across species.

## Results

### Multiple Independent Losses of Condensin II Subunits in Insects

The *Cap-G2* subunit of the condensin II complex is absent in *D. melanogaster* (Herzog et al. 2013), a surprising finding given that in other species, this subunit is necessary for DNA binding and is a target for the regulation of the complex (Yamashita et al. 2011; Piazza et al. 2014). To understand when this loss occurred, we used a three-step BLAST protocol (see Materials and Methods) to search for condensin subunits in dozens of insects, starting with Dipterans and moving outward to more distantly related species. The potential for false negatives in our BLAST-based method does exist, especially for genes in heterochromatin or other regions of poor coverage. But although we cannot have absolute certainty in our absence calls, the phylogenetic signal in our results allows reasonable confidence that the genes we define as “missing” have truly been lost in the taxa in question. The putative losses we identified almost always encompassed several species in a monophyletic manner, even when the quality of the species’ genome assemblies varied substantially.

**Fig. 1.**
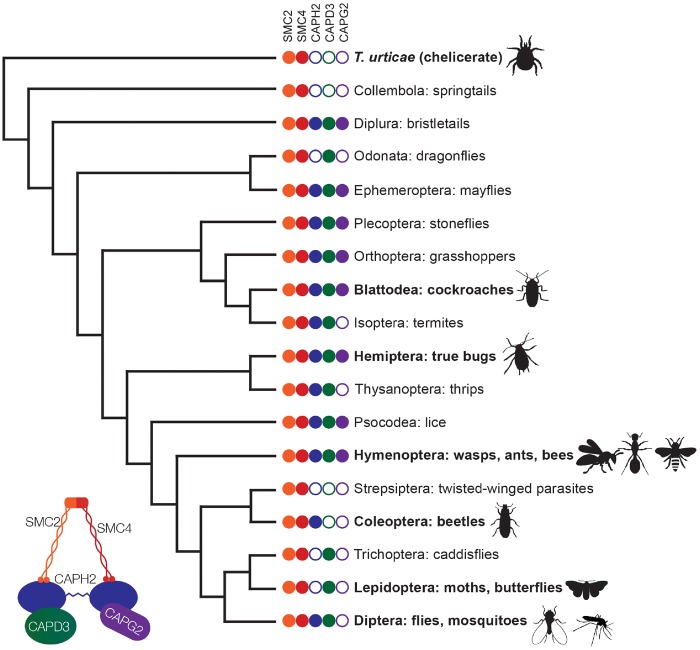
Insect phylogeny shows evidence for multiple independent losses of condensin II subunits. Based on genome sequence analysis, non-SMC subunits have been repeatedly lost in different combinations across several insect orders. Phylogeny is based on Misof et al. (2014) Bold names represent orders containing species where interphase chromosome pairing was also assessed. Condensin II subunits displayed for each order represent all subunits detected in any member of the order; some subgroups within the order have lost further subunits. In all species sampled, we were able to identify all condensin I-specific subunits. This cladogram shows phylogenetic relationships only, and branch lengths do not represent divergence time. For an insect phylogeny with branch lengths to scale and estimates of divergence time provided, see Misof et al. (2014).

We were able to identify all five condensin I subunits in virtually every species we screened, consistent with previous data suggesting that this complex should be conserved in its entirety across eukaryotes (Hirano 2016). When we screened for condensin II subunits, we found that all Dipterans, like *Drosophila*, are missing *Cap-G2*, consistent with previous reports (Herzog et al. 2013). Surprisingly, *Cap-G2* is also absent in several of the orders most closely related to Diptera, including Lepidoptera (butterflies, moths) and Coleoptera (beetles) (fig. 1). The closest relatives of Diptera that retain *Cap-G2* are Hymenoptera (ants, wasps, bees) in which some but not all taxa harbor all five condensin II subunits (supplementary fig. S1, Supplementary Material online). These results suggest that the *Cap-G2* subunit was lost in the ancestor of Diptera over 300 million years ago, and is missing in an astonishing diversity of insects.

Although probing these insect orders, we found that additional subunits of the condensin II complex were also missing in several taxa outside of Diptera. For example, in addition to *Cap-G2*, the *Cap-H2* subunit is also missing in all insects in the orders Lepidoptera and Trichoptera, indicating a loss of this subunit approximately 200 million years ago in these lineages. In Coleoptera, *Cap-D3* has been uniformly lost. Even more dramatically, insects in the order Strepsiptera are missing all non-SMC subunits of condensin II. Depletion of either *Cap-H2* or *Cap-D3* in *Drosophila* results in male sterility (Savvidou et al. 2005; Hartl, Smith, et al. 2008; Hartl, Sweeney, et al. 2008) so the fact that these subunits have been jettisoned in other clades is striking.

When we expanded our study to other insect orders available in the NCBI database, we found further loss events for *Cap-G2*, *Cap-H2*, and *Cap-D3*. Based on parsimony, many of these losses could not have originated from a single common event, and instead must have occurred repeatedly and independently throughout insect evolution. We also identified more taxa where all three non-SMC subunits have been lost: Collembola (springtails), our chelicerate outgroup *Tetranychus urticae* (two-spotted spider mites), and *Trichogramma pretiosum* within Hymenoptera (fig. 1 and supplementary fig. S1, Supplementary Material online). Some of the subunit losses we identified were consistent across every member of an order that we sampled, as in Coleoptera and Lepidoptera (supplementary figs. S2 and S3, Supplementary Material online). We also found cases of more recent and dynamic losses within orders, such as the repeated losses of *Cap-G2* within Hymenoptera (supplementary fig. S1, Supplementary Material online), and isolated *Cap-G2* and *Cap-H2* loss events in Hemiptera (supplementary fig. S4, Supplementary Material online). Although parsimony suggests the common ancestor of insects had a complete condensin II complex, the observed widespread and heterogeneous nature of condensin II subunit losses suggests that these subunits are more dispensable than previously believed.

In species with only one loss, *Cap-G2* is almost always the absent subunit, suggesting that this subunit is often the first to be lost, and is therefore either the most dispensable subunit or the one subject to the strongest evolutionary pressures. Species with further losses complicate existing assumptions about the roles of condensin II subunits. Coleopteran species carry *Cap-H2* but are missing *Cap-G2* and *Cap-D3* (supplementary fig. S2, Supplementary Material online). These HEAT-repeat subunits have crucial roles in DNA binding and loop extrusion (Piazza et al. 2014; Ganji et al. 2018), raising questions about the function of the condensin II complex in their absence. In Lepidoptera, Trichoptera, and Odonata, *Cap-D3* is present but *Cap-H2* and *Cap-G2* are absent (fig. 1 and supplementary fig. S3, Supplementary Material online). Since *Cap-D3* is thought to bind primarily to *Cap-H2* (Herzog et al. 2013; Piazza et al. 2014; Hirano 2016), the nature of the association (if any) between *Cap-D3* and the SMC subunits in these taxa is enigmatic.

In species where condensin II subunits have been lost, we speculated that compensatory duplications could have occurred in the condensin I paralogs of these missing genes. In several example species, we used BLAST to align putative *Cap-G*, *Cap-D2*, and *Cap-H* genes with the full genome of the same species (see Materials and Methods). We did find significant hits in some species, indicating that duplication events had occurred. However, there appeared to be no relationship with condensin II subunit losses: of the condensin I subunit duplications we identified, a majority (9/12) occurred in species where the condensin II paralog was still present, and of species lacking a condensin II component, most (12/14) had no duplication in the condensin I paralog (supplementary file S10, Supplementary Material online). Further, the condensins I and II paralogs within a species almost never align with each other due to their degree of difference, so it seems unlikely that a duplicate of a condensin I gene would evolve to fill the role of a missing condensin II subunit.

### Condensin II Loss Does Not Induce Somatic Homolog Pairing

The condensin II complex antagonizes pairing in *Drosophila* (Hartl, Smith, et al. 2008; Joyce et al. 2012), and *Drosophila* condensin II also lacks the *Cap-G2* subunit (Herzog et al. 2013). Assuming a model in which the prevention of pairing is an active process in many animal genomes (40), we hypothesized that the *Cap-G2* loss in Dipterans could be the cause of a loss of antipairing function and the resultant high levels of pairing observed in flies. Depletion of other condensin II subunits in *Drosophila* is not well tolerated (Hartl, Sweeney, et al. 2008), but our discovery of other insect species with naturally absent condensin II subunits offered us the opportunity to study the relationship between condensin II complex composition and pairing without introducing pleiotropic effects or requiring genetic manipulation.

**Fig. 2.**
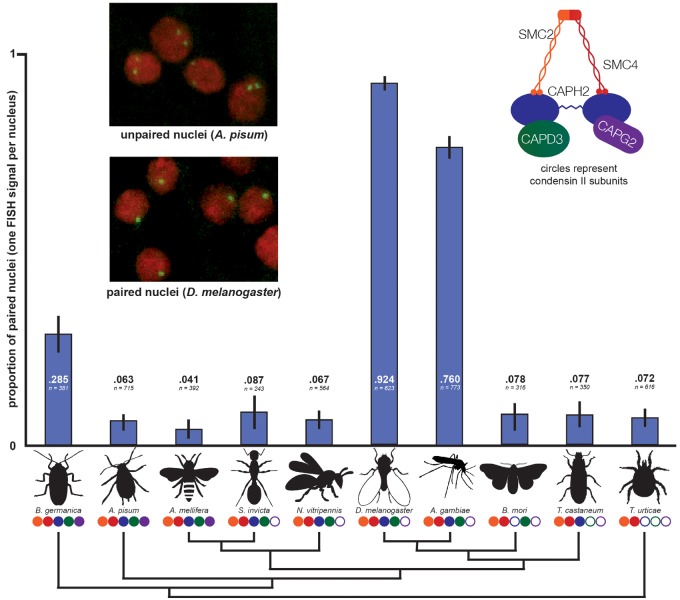
No relationship between condensin II composition and homolog pairing. Species missing more condensin II subunits do not tend to have higher pairing rates as measured by Oligopaint DNA-FISH. Blue bars represent the proportion of nuclei in each species displaying a single FISH signal. Values for each species represent the observed pairing proportion and number of nuclei scored. Each set of values represents results from a single Oligopaint probe. Error bars show 95% confidence intervals (binomial proportion with Wilson score). Circles below species names correspond to condensin II complex composition. Cladogram of insect species, based on the phylogeny of Misof et al. (2014), shows relationships only and is not to scale.

Although it has long been believed that somatic pairing does not occur at high rates in organisms outside of Diptera (White 1954), it has until recently been difficult to measure pairing in other species. In this investigation, we developed custom-designed, species-specific Oligopaint DNA-FISH probes (Beliveau et al. 2012; Beliveau et al. 2018) to target unique sequences, enabling the measurement of pairing behavior at euchromatic loci in non-Dipteran insects. We chose nine insect species and a mite outgroup based on the quality of their genome assemblies, their phylogenetic positions, and the ease of obtaining live specimens. In all these species, we measured pairing levels in interphase nuclei using Oligopaints designed to 300 Kb regions (fig. 2, supplementary fig. S5 and Methods, Supplementary Material online). In five species, we obtained results for two separate Oligopaint probes and found pairing levels to be very similar (supplementary fig. S6, Supplementary Material online). This indicates that the pairing behavior we observed in each species is likely to be representative of the whole genome rather than being locus-specific, consistent with findings in *Drosophila* that pairing levels are similar across nearly all loci, tissues, and life stages (Williams et al. 2007).

In the two Dipterans that we investigated, *D. melanogaster* and *Anopheles gambiae*, over three-quarters of nuclei displayed single FISH signals, indicating high levels of homolog pairing. However, in *Solenopsis invicta* and *Nasonia vitripennis*, which have the same condensin II complex composition as the Dipterans, we observed low-pairing levels (<10% of nuclei). This indicates that *Cap-G2* loss alone does not explain the high pairing levels observed in Dipterans. Next, we expanded our analysis to organisms with different condensin II complex composition. All non-Dipteran species displayed single FISH signals in less than 10% of nuclei, except for *Blattella germanica*, in which 28.5% of nuclei were paired. (This intermediate pairing level may represent a legitimately elevated rate, or be an artifact of higher DNA compaction in the probed region.) The outgroup chelicerate *T. urticae*, which lacks all non-SMC condensin II subunits, displayed low-pairing levels, as did species like *Acyrthosiphon pisum* and *Apis mellifera* with fully intact condensin II complexes. Although condensin II functions as a master regulator and potent antagonist of pairing in *Drosophila*, these results show that most non-Dipteran insect species have the capacity to keep pairing levels minimal regardless of the composition of their condensin II complexes.

### Recurrent Positive Selection of Condensin II in Flies and Mammals

**Fig. 3.**
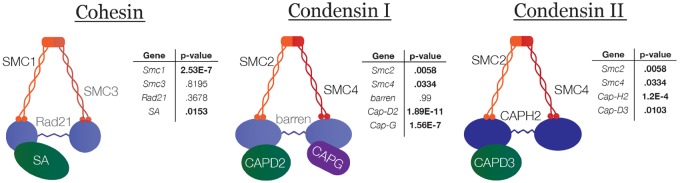
Recurrent positive selection in cohesin and condensin subunits in flies. Results of PAML show widespread positive selection in condensin and cohesin subunits among *Drosophila* species. *P*-values for PAML are derived from a log-ratio test using the log-likelihood scores for the positive selection and neutral models. Using the Bonferroni correction to account for multiple testing, bold values represent statistically significant results with a threshold of *P* = 0.00417.

Our results indicated dynamic evolution of the condensin II complex across long timescales and large phylogenetic distances. To better understand the selective forces driving the evolution of this complex, we investigated whether these changes were accompanied by rapid evolution at the amino acid level in more closely related taxa representing shorter timescales. We first used the McDonald–Kreitman test to detect signatures of recurrent positive selection between *D. melanogaster* and its sister species *Drosophila simulans* (McDonald and Kreitman 1991). In an unpolarized McDonald–Kreitman test using sequences from up to 150 *D. melanogaster* and up to 20 *D. simulans* strains, we detected signatures of positive selection in *Cap-D2* (a component of the condensin I complex), *Cap-D3* (a component of the condensin II complex), *SMC4* (present in both the condensins I and II complexes) and *SMC3* (a component of the cohesin complex) (table 1). These results suggested that the evolution of SMC complexes between these species may be driven by recurrent positive selection, and are consistent with results from previous genome-wide analyses of polymorphisms in *D. simulans* (Begun et al. 2007). To detect signatures of selection in the cohesin and condensin complexes across a wider distribution of *Drosophila* species, we next used a maximum likelihood framework with PAML (Yang 2007). These analyses test for recurrent changes in the sequence of a gene from a distribution of closely related species. In our sample of 17 *Drosophila* species, we found robust signatures of positive selection in nearly all subunits of both condensin complexes and the cohesin complex (fig. 3). To our surprise, almost none of the residues subject to the strongest positive selection were within putative functional domains (supplementary fig. S7, Supplementary Material online). PAML and McDonald–Kreitman tests detect positive selection on different timescales: PAML relies on measuring nucleotide divergence across a group of species and the McDonald–Kreitman test compares polymorphism within species to divergence patterns between a pair of species. Therefore, the variation in results between the two methods is not anomalous. However, the fact that both analyses detect strong positive selection, albeit sometimes in different subunits, is robust evidence for the presence of evolutionary forces driving rapid change in these complexes.

**Table 1.** McDonald–Kreitman Tests Show Positive Selection between *Drosophila melanogaster* and *Drosophila simulans* in *Cap-D2*, *Cap-D3*, *SMC3*, and *SMC4*.

Gene	p*S*	p*N*	d*S*	d*N*	Alpha	*P*-value
Barren (Cap-H)	145	57	33	15	0.1352	0.7237
**Cap-D2**	*565*	*130*	*39*	*19*	*0.5277*	*0.0153*
**Cap-D3**	*544*	*231*	*25*	*53*	*0.7997*	*9.56E-11*
*Cap-G*	*289*	*133*	*60*	*36*	*0.4863*	*0.0155*
*Cap-H2*	99	110	43	59	0.1902	0.3988
*Glu (*SMC4*)*	*278*	*119*	*36*	*30*	*0.4863*	*0.0155*
SA	368	27	48	4	0.1196	0.7723
SMC1	389	18	34	2	0.2133	0.6724
*SMC2*	150	44	24	6	−0.1733	0.9999
**SMC3**	*375*	*5*	*32*	*3*	*0.8506*	*0.0259*

Note.—Italic names represent *P*-values under 0.05 (Fisher’s Exact test, see Materials and Methods). p*S*, polymorphic synonymous changes within species; p*N*, polymorphic nonsynonymous changes within species; d*S*, fixed synonymous changes between species; d*N*, fixed nonsynonymous changes between species.

We hypothesized that these fast-evolving residues could represent binding sites for regulators of the complex. We first considered *SCF^Slmb^*, a ubiquitin ligase that targets *Cap-H2* for degradation (Joyce et al. 2012; Buster et al. 2013). However, an alignment of *Cap-H2* sequences from 18 *Drosophila* species showed that the *SCF^Slmb^* binding motif was well conserved, even though the amino acid sequence was often divergent across the rest of the protein (supplementary fig. S8*A*, Supplementary Material online). This strongly suggests that the pattern of positive selection observed in *Cap-H2* is not driven by pressure to escape *SCF^Slmb^* regulation. Given that the knockdown of *SCF^Slmb^* leads to reduced pairing in *Drosophila* (Joyce et al. 2012), we also speculated that low-pairing insect species might be missing this key regulator. However, BLAST searches revealed that every species used in the pairing assay possessed a putative *SCF^Slmb^* sequence—an unsurprising result given that *SCF^Slmb^* has several other targets besides *Cap-H2*. We also found that the core binding motif for *Mrg15*, an important cofactor for condensin II (Wallace et al. 2015), was well conserved across the same 18 *Drosophila* species (supplementary fig. S8*B*, Supplementary Material online). If interactions with a regulator are driving positive selection in *Cap-H2*, it must be a regulator with an unknown binding motif or identity.

**Fig. 4.**
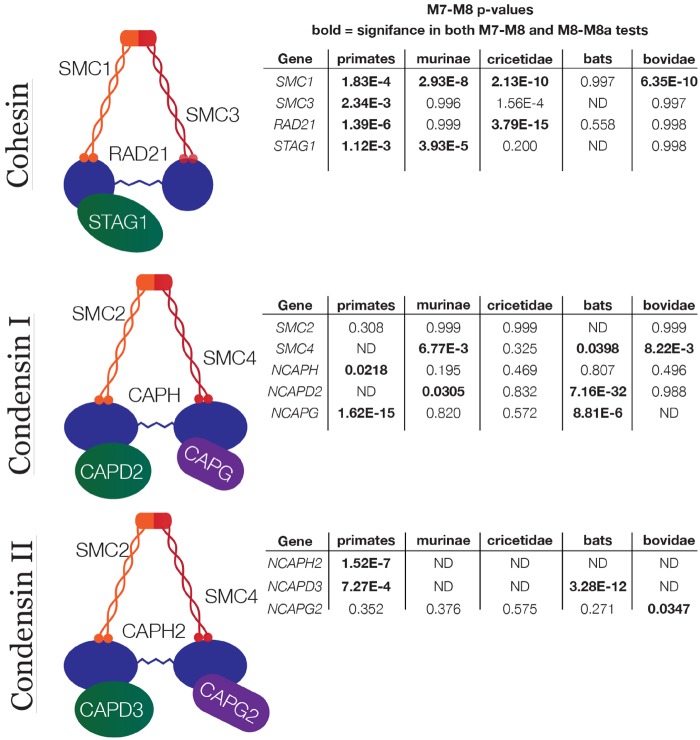
PAML analyses reveal signatures of positive selection in condensins and cohesin in mammal clades. *P*-values are derived from a log-ratio test using the log-likelihood scores for the positive selection and neutral models. Using the Bonferroni correction to account for multiple testing, bold values represent statistically significant results with a threshold of *P* = 0.00417.

To address whether the pattern of rapid amino acid evolution we observed in condensins is present outside of insects, we next conducted PAML analyses across 18 primate species. Our results uncovered robust signatures of recurrent positive selection in cohesin and condensins in primates as well. To ensure that the instances of positive selection we had thus far identified were not anomalous, we gathered sequences for both complexes from a variety of independent mammalian clades and ran PAML on each gene in each clade. Our analysis shows that every one of the mammalian clades we tested has a signature of recurrent, rapid evolution for at least two genes across the complexes (fig. 4). Importantly, none of the genes shown by PAML to be under significant positive selection had recognizable paralogs, indicating that the results were not compromised by duplication events. We were unable to gather sufficient sequence data to analyze every subunit in some species, suggesting that the degree of rapid evolution that we observed may be an underestimate. Together, our results suggest that despite the conserved and essential role of condensins in cell viability, the rapid evolution of these complexes is shaped by positive selection and is a general pattern across a wide variety of organisms.

## Discussion

Condensins are ancient protein complexes that play a fundamental role in genome organization across nearly all cellular life on earth. We show that, even after billions of years of existence, condensin components evolve under recurrent positive selection, and condensin II subunits have experienced rampant losses across orders spanning hundreds of thousands of multicellular eukaryotic species. Although the condensin I complex is ubiquitous in eukaryotes, the composition of the condensin II complex appears to be evolutionarily labile. The absence of the *Cap-G2* subunit in Diptera initially appeared anomalous, but our results show that the *Cap-G2* subunit loss dramatically predates the origin of Diptera, and encompasses taxa representing nearly half of all described species on earth. Our results further suggest that many orders of organisms are missing the condensin II complex altogether. These losses raise questions about the mechanisms of genome organization in these taxa and across eukaryotes.

In particular, our data debunk the conjecture that condensin II complex composition directly determines the degree of pairing in an organism. Instead, our results indicate that somatic homologous chromosome pairing is a Dipteran-specific innovation, confirming previous speculation (White 1954). These findings have several important implications. First, a powerful and well-established functional output of somatic pairing is gene regulation through the *trans* action of regulatory elements (Wu and Morris 1999; Joyce et al. 2016). To the extent that transregulation depends on close pairing of homologous chromosomes, our results predict that transvection should be limited to Dipteran species as well. Second, according to an emerging viewpoint, the pairing of homologous chromosomes may be an inevitable consequence of DNA sequence homology, and non-Dipteran species may expend considerable effort to keep homologs separate in somatic cells (Joyce et al. 2016). Under this scenario, our results suggest that non-Dipteran species utilize as yet undiscovered condensin II-independent mechanisms to separate homologous chromosomes. Alternatively, if chromosome pairing is an active process, our results raise the question of how and why such a drastic change in global nuclear organization has evolved in Dipterans.

The signatures of recurrent positive selection we observed in the condensin and cohesin complexes across *Drosophila* and mammals suggest that the evolution of these SMC complexes is shaped by evolutionary arms races driven by genetic conflict. Previous findings regarding the role of condensins support this view. First, the condensin II complex is known to be enriched around rDNA (Schalbetter et al. 2017) and pericentromeric heterochromatin (Nishide and Hirano 2014). These regions are important for proper pairing and chromosome segregation in *Drosophila*, suggesting that the evolution of these complexes may be influenced by the dynamics of female meiotic drive, as is the case with centromeric histone H3 variant *Cid* (Malik and Henikoff 2001). Second, mutants of the condensin II complex genes in Drosophila are viable but male sterile (Hartl, Sweeney, et al. 2008; Hirano 2016), indicating their essential role in male meiosis. In *Drosophila*, chromosome decondensation is often observed in the germlines of sterile interspecies hybrid males and males carrying naturally occurring segregation distorters, raising the possibility that the condensin II complex has evolved under pressure from segregation distorters. Third, the condensin II complex has been shown to localize to retrotransposon sequences and mediate their repression in flies (Schuster et al. 2013) and humans (Ward et al. 2017). As genomes continually evolve to suppress retrotransposon sequences, intragenomic conflicts involving transposable elements may also drive positive selection in *Cap-D3*, and potentially the rest of the condensin II complex. Finally, in addition to intragenomic conflict, host-pathogen dynamics may also drive the evolution of condensins. In humans, *SMC4* regulates the innate immune response, and in *Drosophila*, *Cap-D3* responds to bacterial infection by up-regulating the expression of antimicrobial peptides (Longworth et al. 2012; Wang et al. 2018). Epstein–Barr virus, the causative agent of mononucleosis, is also known to activate *Cap-G* to force compaction of the host genome (Lee et al. 2007). Given the evolutionary pressures acting on the condensin complexes both from inside and outside the genome, these complexes may be positioned at the crossroads of several forms of evolutionary conflicts.

These evolutionary conflicts may not only explain the patterns of positive selection that we observed in condensin, but also the repeated loss of condensin II subunits. Though the condensin II complex plays critical roles in genome organization, other genes may have evolved to take over some of these functions when conflicts have forced the abandonment of condensin II subunits. Condensin I could simply act as a jack-of-all-trades, as it does in yeast, bacteria, and archaea, or nonorthologous machinery may replace some condensin II functionality when it is lost. This notion is consistent with reports of varying cellular responses to depletion of condensins across different cell types, cell lines, and species (Fazzio and Panning 2010; Green et al. 2012; Nishide and Hirano 2014). Although the specific nature of this functional replacement is speculative, some redundancy would allow taxa to jettison condensin II subunits entirely to side-step evolutionary conflicts.

Taken together, our work shows that the evolution of condensin subunits is more dynamic than previously suspected, and their function is more elastic. Our findings highlight the value of interrogating the evolution of these deeply conserved genes, and of exploring their function in nonmodel organisms. Despite the great heterogeneity of condensin II complex composition, we observed that non-Dipteran insects consistently organize their genomes without chromosome pairing, whereas Dipterans have taken a diametrically opposite path of near complete homologous chromosome pairing. Our results open the door to further investigations to elucidate the factors that underlie the mechanics of genome organization across the diversity of life.

## Materials and Methods

### Inferring Condensin II Subunit Losses

To characterize condensin II composition across *Insecta*, we sampled 97 species: 3 Dipterans, 18 Lepidopterans, 9 Coleopterans, 40 Hymenopterans (16 Formicoidea, 11 Apoidea, and 13 other), 13 Hemipterans, and 14 members of other orders, using 2 species per order where genomes existed. Initial searches were conducted in February–May 2017, and results were manually double-checked in November 2018. For all species, we used the NCBI TBlastN function (Altschul et al. (1990)) to search through each insect genome, following a three-step protocol. First, we gathered canonical publicly available sequences for all condensins I and II subunits in *D. melanogaster* (downloaded from FlyBase, based on the Release 6 genome) and *Homo sapiens* sequences (from UniProt, assembly GRCh38.p12). We searched with *D. melanogaster* and *H. sapiens* sequences against both nucleotide collection (nr/nt) and whole-genome shotgun contigs (wgs) for each target species. This initial search yielded predicted condensin II subunit sequences for species within most insect orders. Second, we selected subunits from at least one species with an annotated genome to use as secondary bait for species within that order. For orders where we were unable to identify annotated genes corresponding to specific subunits, we used sequences from the most closely related orders. Third, in cases where a species had a putative subunit loss but had within-order relatives that retained the subunit, we used the subunit sequence for the most closely related species as a tertiary bait to probe the target species genome. If any of these three steps yielded a hit, we deemed the subunit to be present, and if all three failed to produce a hit, we considered the subunit to be absent. In all cases, we used the default BLAST search parameters (BLAST+ version 2.8.0-alpha), except that we searched against whole-genome shotgun contigs in species lacking genome annotations. For strong hits with E-values of 1E-10 or less with multiple regions of alignment, we considered the subunit to be present without further validation. We validated weaker hits (E-values between 1E-10 and 0.05 or only a single region of alignment) by BLASTing the putative sequence against annotated genomes of related species and confirming that our sequence aligned with the expected subunit. See supplementary file S9, Supplementary Material online, for accession numbers of identified subunits in example species. All alignment files are deposited in the Dryad repository (doi: 10.5061/dryad.4m6j54g).

Demonstrating the absence of a subunit is a difficult task, and we cannot eliminate the possibility of false negatives, especially in phylogenetically isolated species. However, the condensin I complex functioned as a reliable control. In all species in our phylogenies, we were readily able to identify all five condensin I subunits, suggesting that our method was able to identify subunits where they were present. Importantly, there is little homology between condensin I subunits and their condensin II paralogs: pairs of paralogs are rarely hit with the same bait, and when they are, one hit is always of much greater quality. (See supplementary file S10, Supplementary Material online, for examples.) As we show, condensin II subunits are undergoing rapid evolution in many taxa, so it is formally possible that these subunits have diverged to the point of being undetectable by our alignment-based method even as their condensin I paralogs remain apparent. However, the condensin II hits we did generate were generally just as robust as those for condensin I subunits, suggesting that rapid evolution is unlikely to have contributed to systematic false negatives in our presence/absence analysis.

Further, our method uncovered identical condensin II complex composition within several taxa despite substantial variation in genome assembly quality. For example, all 11 species investigated within Apoidea had lost only *Cap-G2*, all 16 Formicoidea species had every subunit represented, and *Cap-H2* and *Cap-G2* are missing in all 18 of the Lepidopteran species sampled (supplementary figs S1 and S3, Supplementary Material online). Even when condensin II complex composition within a clade was not uniform, the putative losses occurred in a phylogenetically consistent manner, thus providing further confidence in our conclusions.

### Inferring Condensin I Subunit Duplications

We explored the possibility of condensin I subunit duplications for 12 species: every species used in the pairing analysis except *B. germanica*, plus the Isopteran *Zootermopsis nevadensis* and the Hemipterans *Nilaparvata lugens* and *Diaphorina citri*. We BLASTed putative *Cap-H*, *Cap-D2*, and *Cap-G* subunit sequences for each species against the species’ own genomes using the default settings. All hits with E-values below 0.001 were then filtered. First, we eliminated hits with large areas (>200 nt) of near-perfect (>98%) identity, reasoning that these likely represented sequencing artifacts rather than legitimate duplications. Next, unless the hit was already annotated as a condensin I subunit paralog, we BLASTed hits against close relative species, and eliminated those for which the appropriate condensin I subunit was not the best match. In two species, when *Cap-D2* was used as bait, *Cap-D3* was hit. Beyond this, the condensin II paralogs of condensin I subunits were not hit by this method. (See supplementary file S11, Supplementary Material online, for detailed duplication results. Duplication results are deposited in the Dryad repository doi: 10.5061/dryad.4m6j54g.) Although we did not formally test for condensin II subunit duplications, anecdotally, we did not observe patterns of multiple genes being hit in our BLAST presence/absence investigation. We saw only one unannotated noncondensin gene hit in 26 example searches—see “Other Orders” in alignments folder and Duplications folder in the Dryad repository (doi: 10.5061/dryad.4m6j54g).

### Tests for Positive Selection

Initial evidence for positive selection among condensin and cohesin subunits in *Drosophila* came from an unpolarized McDonald–Kreitman test using up to 150 *D. melanogaster* sequences and up to 20 *D. simulans* sequences obtained from PopFly (Hervas et al. 2017). We found that the rate of nonsynonymous substitutions (d*N*/d*S*) between *D. melanogaster* and *D. simulans* is significantly elevated over the null expectation in *SMC3*, *Cap-D2*, and *Cap-D3*. To validate these results, further analyses for signatures of positive selection were conducted according to the method described in Cooper and Phadnis (2017). Briefly, we analyzed rates of evolution for all condensin and cohesin complex subunits in 17 species of *Drosophila*, 16 species of primates, and 6–14 species within other mammal clades. We conducted analyses for signatures of positive selection using PAML, and tested for recurrent positive selection by comparing NSsites models M7 (neutral) and M8 (positive selection) with 0 as the branch model. We present the *P*-value of the log-ratio test using the log-likelihood scores for the two models. We have not applied a multiple testing correction to our results, because it is not clear that analyzing different subunits represents multiple testing of the same hypothesis. This is concordant with the methods in previous studies using McDonald–Kreitman tests and PAML analysis to detect positive selection in protein complexes (Presgraves and Stephan 2007; Daugherty et al. 2014; Cooper and Phadnis 2017). See supplementary fig. S9, Supplementary Material online, for maximum d*S*, average d*N*/d*S*, and maximum d*N*/d*S* plots for all mammal clades. Data relating to positive selection analysis, including the PAML control file, all alignments, species lists, and max d*S* and average d*N*/d*S* information, is deposited in the Dryad repository (doi: 10.5061/dryad.4m6j54g).

### Oligopaint Probe Design

Oligopaint probes were designed to each species using the OligoMiner pipeline (Beliveau et al. 2018). In brief, we retrieved genomic assemblies or contigs from NCBI Genome database, and built genome indices using Bowtie2 (supplementary file S12, Supplementary Material online). Default settings of the OligoMiner scripts were used to mine these sequences for oligos, except for changing the length requirement to 50mers. FISH targets were chosen based on 300 kb windows with the highest density of oligos.

### Slide Preparation, FISH Protocol, and Microscopy

DNA-FISH was conducted on tissues from ten arthropod species according to a protocol adapted from Larracuente and Ferree (2015). Pairing was then quantified by scoring FISH signals. See Supplementary Material online, for details and for insect husbandry information.

## Supplementary Material

Supplementary data are available at *Molecular Biology and Evolution* online.

## Supplementary Material

msz140_Supplementary_Data
