# Supplementary material for: Recurrent Losses and Rapid Evolution of the Condensin II Complex in Insects
Source: Mol Biol Evol. 2019 Jul 4;36(10):2195–204. doi: 10.1093/molbev/msz140 (PMC6759200; doi:10.1093/molbev/msz140)
Supplement: msz140_Supplementary_Data [file msz140_supplementary_data.zip › Condensin MBE revision 3 supplement.docx]

**Supplementary Materials**

**Recurrent losses and rapid evolution of the condensin II complex in insects**

**King, T. D.^1^, Leonard, C.J. ^1^, Cooper, J.C. ^1^, Nguyen, S. ^2^, Joyce, E.** ^2^, and Phadnis, N.** ^1^**

**Supplementary Methods**

***Insect Husbandry***

***Acyrthosiphon pisum* (pea aphid):** Aphids were obtained from Carolina Biological Supply Company (Burlington, NC) and maintained at 21°C on fava bean (*Vicia faba*) plants. Slides were prepared using whole heads of adults.

***Anopheles gambiae*:** Live mosquito larvae were obtained from Michael Povelones (UPENN) and maintained at 21ºC in tap water. Slides were prepared from brains and head tissue of third- and fourth-instar larvae.

***Apis mellifera* (Western honeybee):** Bees were sourced from BeeWeaver Apiaries (Navasota, TX) and were maintained at 21°C and provided with honey and water. Slides were prepared from dissected brains and head tissue of adult females.

***Blattella germanica* (German cockroach):** Cockroaches were obtained from Carolina Biological Supply Company (Burlington, NC) maintained at 28°C, and provided with water, potato slices, Blue brand dog treats, and Purina brand dry kitten food. Slides were prepared from brains and head tissue of adults.

***Bombyx mori* (silkworm):** Silkworms were obtained from Mulberry Farms (Fallbrook, CA), maintained at 28ºC, and fed Powdered Silkworm Chow (Mulberry Farms). Slides were prepared from brains and head tissue of third- and fourth-instar larvae.

***Drosophila melanogaster*:** All flies used were from a standard *w^1118^* stock, and were maintained at 21 ºC on standard *Drosophila* media. Slides were prepared from a mixture of imaginal discs and brains of third-instar larvae to enrich for diploid nuclei.

***Nasonia vitripennis*:** *Nasonia* cultures were grown with *Calliphoridae* pupae as a host and maintained at 18 ºC. Slides were prepared from whole heads of adult females.

***Solenopsis invicta* (fire ant):** Ants were obtained from a wild Florida colony, maintained at 28ºC, provided with water, and fed with silkworms, aphids, *Drosophila*, and Purina brand dry cat food. Slides were prepared from brains and head tissue of adult females.

***Tribolium castaneum* (red flour beetle):** Beetles were obtained from Carolina Biological Supply Company and maintained at 21 ºC in a 20:1 mix of white flour and brewer’s yeast with carrot pieces. Slides were prepared from brains and head tissue of adults.

***Tetranychus urticae* (two-spotted spider mite):** Mites used were from the *Sh-Co* strain, which were isolated by Richard Clark while imbibing coffee from the Sugarhouse Coffee Company, SLC, UT (therefore named *Sh-Co*). Mites were maintained at 25 ºC on kidney bean (*Phaseolus vulgaris*) and fava bean (*Vicia faba*) leaves. Slides were prepared from whole female juveniles and adults.

***Slide preparation and FISH protocol***

DNA-FISH was conducted according to a protocol adapted from Larracuente and Ferree (Larracuente and Ferree 2015). Tissues specified in the preceding section were dissected in 1X PBS at room temperature. For all species, tissues were dissected from living or freshly sacrificed animals, and each batch used tissues from at least three animals. In *Drosophila,* cells from life stages and tissues other than early embryo display similar pairing levels (Williams et al. 2007), so we did not rigorously control the developmental stage or tissues analyzed between species, provided that the tissues were diploid. Immediately after dissection, tissues were placed in PBST (PBS w/ 0.1% (v/v) Triton) on ice for 5-30 minutes. Tissues were then incubated for 5-10 minutes in 0.5M sodium citrate on ice. Fixation was performed for 5 minutes directly on poly-L-lysine-coated slides using between 20 μl and 40 μl of fixative solution composed of 3:1 45% acetic acid solution and fresh 16% paraformaldehyde solution (Life Biotechnologies, Carlsbad, CA). Coverslips siliconized with SigmaCote (Sigma-Aldrich, St. Louis, MO) were pressed onto the slides firmly by hand to squash the tissues. Slides were then immediately immersed in liquid nitrogen. After immersion, coverslips were removed, and slides were incubated in cold 100% ethanol for 5 minutes. Slides were then allowed to air-dry completely. FISH hybridization buffer (FHB) was composed of 50% (v/v) 2X SSCT (SSC w/ 0.1% Tween) and 50% formamide with 20% (w/v) dextran sulfate, and stored at 4 ºC. For each round of FISH, 1 μl of 10 mg/ml RNase A solution and1 μl and 3 μl of each Oligopaint probe (depending on probe reliability) were added to 25-40 μl of FHB (depending on tissue volume). The complete FHB and probe mix was then placed on coverslips and added to the slides. Slides were then incubated on a heat block at 90.7 ºC for 7 minutes, sealed with Parafilm, and placed overnight in a humidified chamber at 37 °C. After hybridization, slides were washed in 2X SSCT for 15 minutes, in 0.1X SSC for 15 minutes, and in 0.1X SSC with 0.1% (v/v) DNA stain (either DAPI or TOPRO-3) for 15 minutes. Slides were then air-dried, mounted with Vecta-Shield media (Vector Laboratories, Burlingame, CA), and sealed with nail polish.

***Microscopy and quantitative measures of homolog pairing***

All images were acquired using a Zeiss LSM 880 Airy Scan confocal microscope. For all species, at least 5 images were acquired from areas enriched for diploid cells displaying strong signal. In *B. mori*, *S. invicta*, and *T. castaneum*, two separate slides were imaged and scored. Images were processed using ImageJ software and scored manually. Nuclei were scored as having zero, one, two, or three or more FISH signals, and a separate category for grossly polyploid nuclei was tracked. Multiple signals were judged to exist if multiple peaks of FISH intensity were distinguishable by eye, even if the edges of these signals overlapped. The pairing proportion was defined as the number of nuclei displaying one signal divided by the sum of one-signal and two-signal nuclei. Slides or regions containing high proportions of zero-signal or abnormal nuclei were excluded from analysis.

**Supplementary References**

S1. Peters RS, et al. (2017) Evolutionary History of the Hymenoptera. *Curr Biol* 27(7):1013–1018.

S2. Quicke D, van Achterberg C (1990) *Phylogeny of the subfamilies of the family Braconidae (Hymenoptera: Ichneumonoidea)* (Zoologische Verhandlingen, Leiden).

S3. Zhang S-Q, et al. (2018) Evolutionary history of Coleoptera revealed by extensive sampling of genes and species. *Nat Commun* 9(1):205.

S4. Regier JC, et al. (2013) A Large-Scale, Higher-Level, Molecular Phylogenetic Study of the Insect Order Lepidoptera (Moths and Butterflies). *PLoS One* 8(3):e58568.

S5. Freitas AVL, Brown KS (2004) Phylogeny of the Nymphalidae (Lepidoptera). *Syst Biol* 53(3):363–383.

S6. Kozak KM, et al. (2015) Multilocus Species Trees Show the Recent Adaptive Radiation of the Mimetic Heliconius Butterflies. *Syst Biol* 64(3):505–524.

S7. Zakharov E V., Caterino MS, Sperling FAH (2004) Molecular Phylogeny, Historical Biogeography, and Divergence Time Estimates for Swallowtail Butterflies of the Genus Papilio (Lepidoptera: Papilionidae). *Syst Biol* 53(2):193–215.

S8. Song N, Liang A-P, Bu C-P (2012) A Molecular Phylogeny of Hemiptera Inferred from Mitochondrial Genome Sequences. *PLoS One* 7(11):e48778.

S9. Nováková E, et al. (2013) Reconstructing the phylogeny of aphids (Hemiptera: Aphididae) using DNA of the obligate symbiont Buchnera aphidicola. *Mol Phylogenet Evol* 68(1):42–54.

S10. Schultz J, Milpetz F, Bork P, Ponting CP (1998) SMART, a simple modular architecture research tool: identification of signaling domains. *Proc Natl Acad Sci U S A* 95(11):5857–64.

S11. Obbard DJ, et al. (2012) Estimating Divergence Dates and Substitution Rates in the Drosophila Phylogeny. *Mol Biol Evol* 29(11):3459–3473.

S12. Edgar RC (2004) MUSCLE: multiple sequence alignment with high accuracy and high throughput. *Nucleic Acids Res* 32(5):1792–1797.

**Figure Legends**

**Figure S1. Hymenopteran phylogeny shows independent within-order losses of CapG2.** Species phylogeny is based on Peters et al. (Peters et al. 2017) with distinctions within Ichneumonoidea based on Quicke and van Achterberg (S2). Bold names represent species or clades in which pairing was assessed. Red lines indicate inferred independent losses of condensin II subunits. All condensin I subunits are present in all species shown. Cladogram shows phylogenetic relationships only and is not to scale.

**Figure S2. Coleopterans show consistent patterns of CapD3 and CapG2 loss.** All condensin I subunits are present in all species shown. Cladogram is based on the phylogeny of Zhang et al. (S3), shows phylogenetic relationships only, and is not to scale.

**Figure S3. Lepidopterans show consistent CapH2 and CapG2 loss.** Whole-order phylogeny based on Regier et al (S4), with Nymphalidae based on Freitas and Brown (S5), Heliconius based on Kozak et al. (S6), and Papilio based on Zakharov et al. (S7). All condensin I subunits are present in all species shown. Cladogram shows phylogenetic relationships only and is not to scale.

**Figure S4. Hemipteran phylogeny shows independent loss events of CapG2 and CapH2.** All condensin I subunits are present in all species shown. Cladogram is based on the species phylogeny of Song et al. (S8), with distinctions within Aphididae based on Nováková *et al*. (S9), and is not to scale.

**Figure S5. Representative FISH images.** For all images, red represents DNA stain, and green represents the Oligopaint probe channel. All images are at the same scale, and the scale bar in panel H represents 5 μm. Images A-J are from the main probes used for each species, and images K-O show results from the second probe, when one was used. DNA in images A-E and I-N is stained with DAPI, and DNA in images F-H and O is stained with TOPRO-3. Image A shows the probe NP17, labeled with Cy3, in *A. mellifera* tissue. Image B shows NP01 (Cy3) in *A. pisum*, C shows NP04 (Cy5) in *B. germanica*, D shows NP05 (Cy3) in *B. mori*, E shows Null4 (Cy5) in *D. melanogaster*, F shows NP09 (Cy3) in *N. vitripennis*, G shows NP11 (Cy3) in *S. invicta*, H shows NP13 (Cy3) in *T. castaneum*, I shows NP16 (Cy5) in *T. urticae*, and J shows 231 (Cy3) in *A. gambiae*. For alternate probes, K shows NP18 (6-FAM) in *A. mellifera*, L shows NP02 (Cy5) in *A. pisum*, M shows 232 (Cy5) in *A. gambiae*, N shows NP06 (Cy5) in *B. mori*, and O shows NP10 (6-FAM) in *N. vitripennis*. In all images, white arrows indicate examples of nuclei scored as paired (if present), yellow arrows indicate examples of unpaired nuclei, and blue arrows indicate examples of nuclei excluded from calculations of pairing proportions due to apparent polyploidy, lack of signal, indeterminate nuclear boundaries, or poor signal-to-noise ratio (see Materials and Methods).

**Figure S6. Pairing data for alternate probes indicates little within-species variation.** In *A. pisum*, *A. mellifera*, *N. vitripennis*, *A. gambiae,* and *B. mori*, results were obtained for both Oligopaint probes synthesized. Results for the probes with worse signal or fewer nuclei scored in each species were excluded from the main analysis, but are reported here. Main probe results are shown in blue, and alternate probe results in green. Values for each species represent the observed pairing proportion and number of nuclei scored. Error bars show 95% confidence intervals (binomial proportion with Wilson score). Proportion of nuclei with single FISH signals observed with the alternate probes was highly consistent with that observed in main probe, except in *A. mellifera*.

**Figure S7. Sites of repeated positive selection in condensin and cohesin subunits.** Red arrows show residues subject to positive selection (dN/dS) across insect lineages, as indicated by PAML. Sites under selection were identified by the Bayes-EmpiricalBayes test in PAML, with a posterior-probability >0.90. Boxes indicate putative functional domains as identified by SMART (S10).

**Figure S8. Cap-H2 regulator binding motifs show strong conservation across *Drosophila*.** Cap-H2 sequences of 18 *Drosophila* species representing ~40 million years of evolutionary divergence were aligned with MUSCLE software and visualized with Jalview (S11, S12). Darker blue represents more highly conserved residues. (A) We found high conservation relative to the surrounding region in the SLMB binding motif (DSGISS, outlined in red). (B) We also observed high conservation in the Mrg15 binding motif (FKLP, in red).

**Figure S9. Amino acid substitution rates for mammal clades used in PAML.** Plots show the distributions of average dN/dS, maximum dN/dS, and maximum dS for all species sampled in each of the mammal clades used in PAML.

**File S10. Condensin BLAST master list.** This spreadsheet contains accession numbers for putative condensin I and II subunits for example species from each order, as well as all Hemipterans and Coleopterans. Where the indicated condensin subunits were present, accession numbers are given for genes in the case of annotated genomes, or contigs containing the hit in unannotated ones. "No" indicates that the indicated subunits was searched for using the BLAST protocol described in Materials and Methods but not found. In some species, data is not shown for all subunits. Blank cells indicate that the accession number is not shown, and do not necessarily indicate a lack of the subunit. For full alignments for all subunits, see File S12.

**File S11. Condensin duplication analyses.** This spreadsheet contains results from our searches for potential duplication events of condensin I subunits in 12 example species representing seven orders, including accession numbers for putative duplicates.

**File S12. Probe information compilation.** This spreadsheet contains information on the contig, genomic location, probe count, and probe density for Oligopaint probes used in the pairing assay.

***The following items are hosted in the Dryad repository (doi:10.5061/dryad.4m6j54g):***

**Condensin BLAST alignments.** This folder contains full results and alignments for BLAST searches for condensin II subunits. Data is subdivided by order, with additional subsections for condensin subunit sequences from species used as bait. “Lost subunits” subsection indicates subunits that were missed in the initial searches performed with standard bait sequences and were redone with close relative sequences, sometimes revealing hits.

**Duplication alignments.** This folder contains full results and alignments for BLAST searches looking for compensatory duplications in condensin I subunits.

**Condensin PAML.** This folder contains data relating to positive selection analysis, including the PAML control file, all alignments, species lists, and max dS and average dN/dS information.

**Figure S1. Hymenopteran phylogeny shows independent within-order losses of CapG2.** Species phylogeny is based on Peters et al. (Peters et al. 2017) with distinctions within Ichneumonoidea based on Quicke and van Achterberg (S2). Bold names represent species or clades in which pairing was assessed. Red lines indicate inferred independent losses of condensin II subunits. All condensin I subunits are present in all species shown. Cladogram shows phylogenetic relationships only and is not to scale.


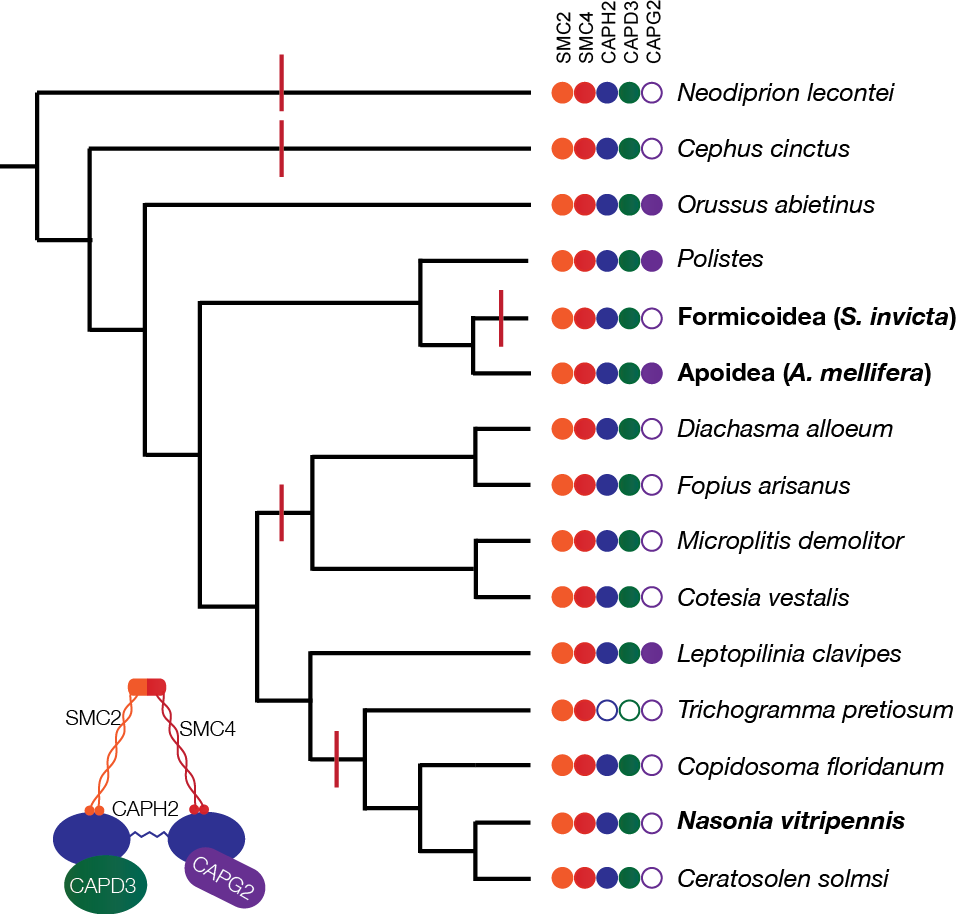


**Figure S2. Coleopterans show consistent patterns of CapD3 and CapG2 loss.** All condensin I subunits are present in all species shown. Cladogram is based on the phylogeny of Zhang et al. (S3), shows phylogenetic relationships only, and is not to scale.


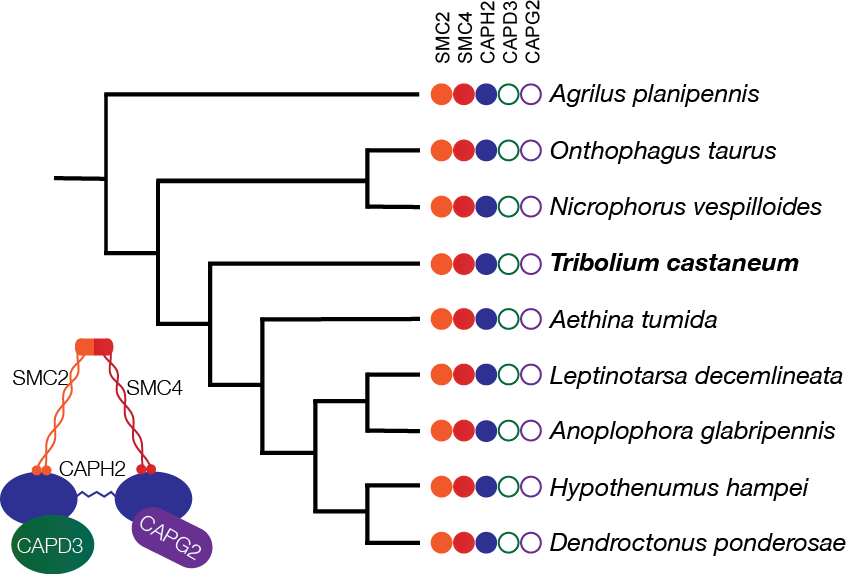


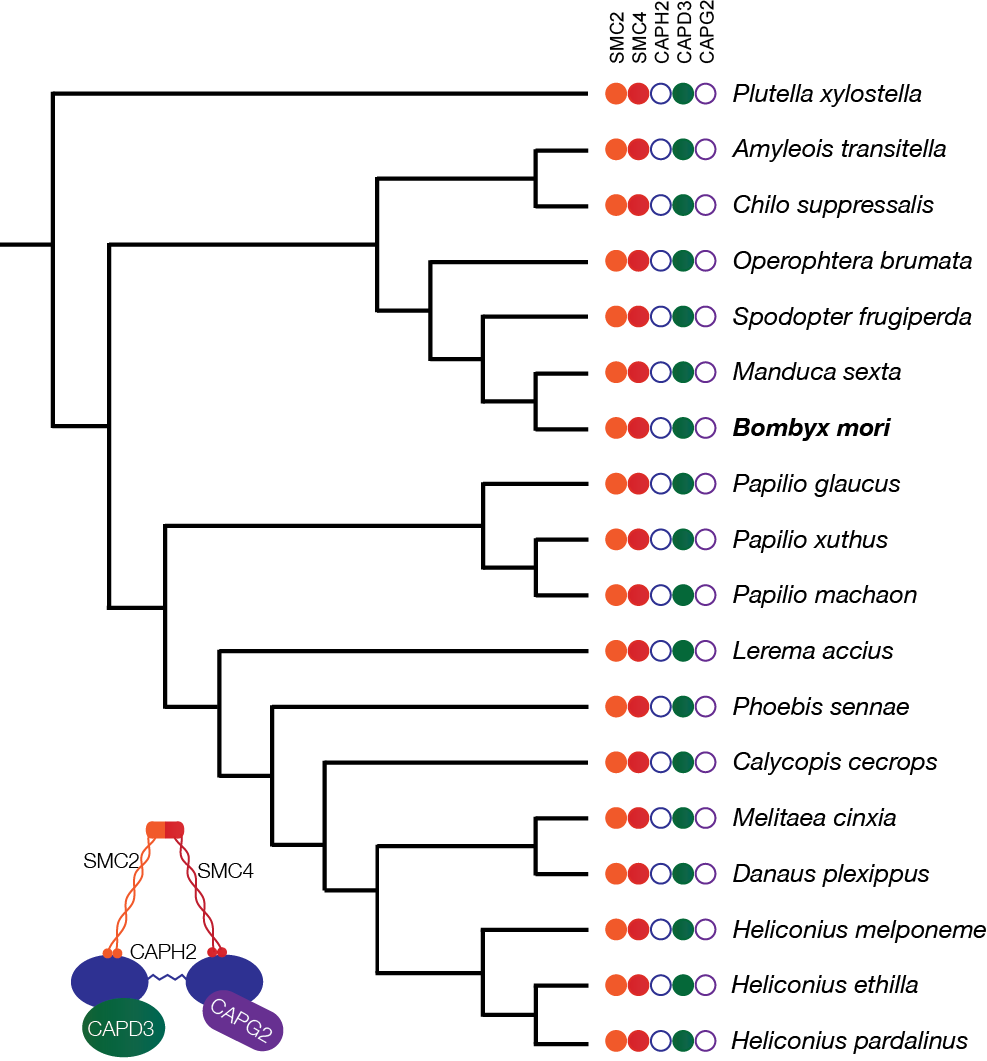


**Figure S3. Lepidopterans show consistent CapH2 and CapG2 loss.** Whole-order phylogeny based on Regier et al (S4), with Nymphalidae based on Freitas and Brown (S5), Heliconius based on Kozak et al. (S6), and Papilio based on Zakharov et al. (S7). All condensin I subunits are present in all species shown. Cladogram shows phylogenetic relationships only and is not to scale.

**Figure S4. Hemipteran phylogeny shows independent loss events of CapG2 and CapH2.** All condensin I subunits are present in all species shown. Cladogram is based on the species phylogeny of Song et al. (S8), with distinctions within Aphididae based on Nováková *et al*. (S9), and is not to scale.


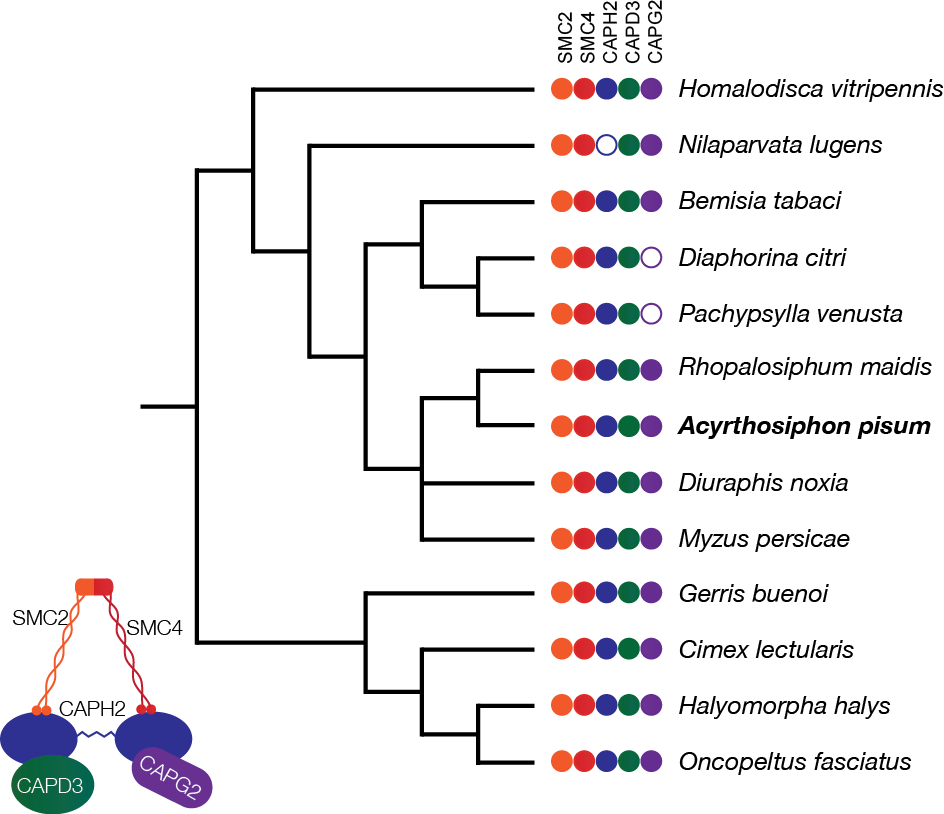


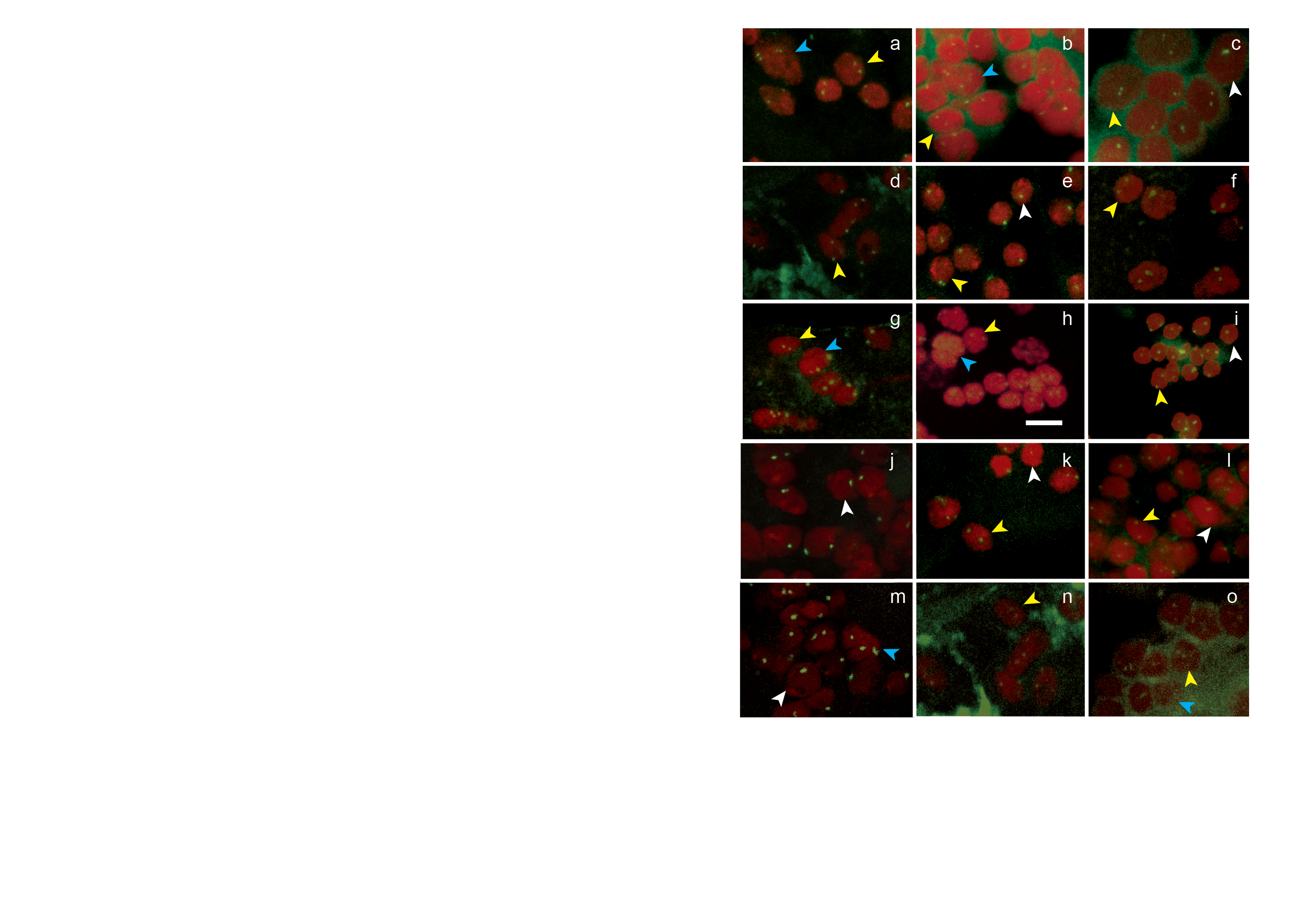


**Figure S5. Representative FISH images.** For all images, red represents DNA stain, and green represents the Oligopaint probe channel. All images are at the same scale, and the scale bar in panel H represents 5 μm. Images A-J are from the main probes used for each species, and images K-O show results from the second probe, when one was used. DNA in images A-E and I-N is stained with DAPI, and DNA in images F-H and O is stained with TOPRO-3. Image A shows the probe NP17, labeled with Cy3, in *A. mellifera* tissue. Image B shows NP01 (Cy3) in *A. pisum*, C shows NP04 (Cy5) in *B. germanica*, D shows NP05 (Cy3) in *B. mori*, E shows Null4 (Cy5) in *D. melanogaster*, F shows NP09 (Cy3) in *N. vitripennis*, G shows NP11 (Cy3) in *S. invicta*, H shows NP13 (Cy3) in *T. castaneum*, I shows NP16 (Cy5) in *T. urticae*, and J shows 231 (Cy3) in *A. gambiae*. For alternate probes, K shows NP18 (6-FAM) in *A. mellifera*, L shows NP02 (Cy5) in *A. pisum*, M shows 232 (Cy5) in *A. gambiae*, N shows NP06 (Cy5) in *B. mori*, and O shows NP10 (6-FAM) in *N. vitripennis*. In all images, white arrows indicate examples of nuclei scored as paired (if present), yellow arrows indicate examples of unpaired nuclei, and blue arrows indicate examples of nuclei excluded from calculations of pairing proportions due to apparent polyploidy, lack of signal, indeterminate nuclear boundaries, or poor signal-to-noise ratio (see Materials and Methods).

**Figure S6. Pairing data for alternate probes indicates little within-species variation.** In *A. pisum*, *A. mellifera*, *N. vitripennis*, *A. gambiae,* and *B. mori*, results were obtained for both Oligopaint probes synthesized. Results for the probes with worse signal or fewer nuclei scored in each species were excluded from the main analysis, but are reported here. Main probe results are shown in blue, and alternate probe results in green. Values for each species represent the observed pairing proportion and number of nuclei scored. Error bars show 95% confidence intervals (binomial proportion with Wilson score). Proportion of nuclei with single FISH signals observed with the alternate probes was highly consistent with that observed in main probe, except in *A. mellifera*.


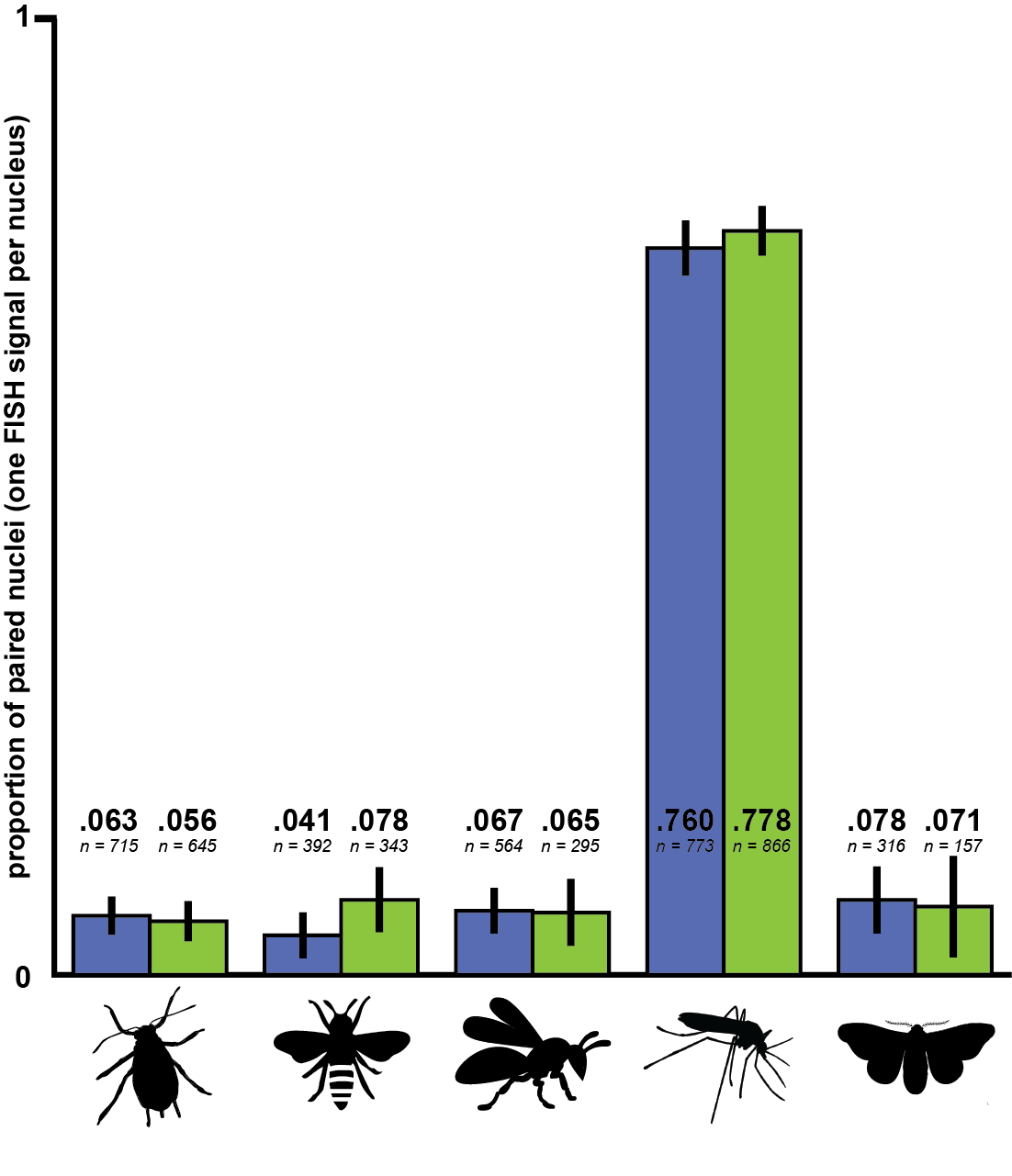

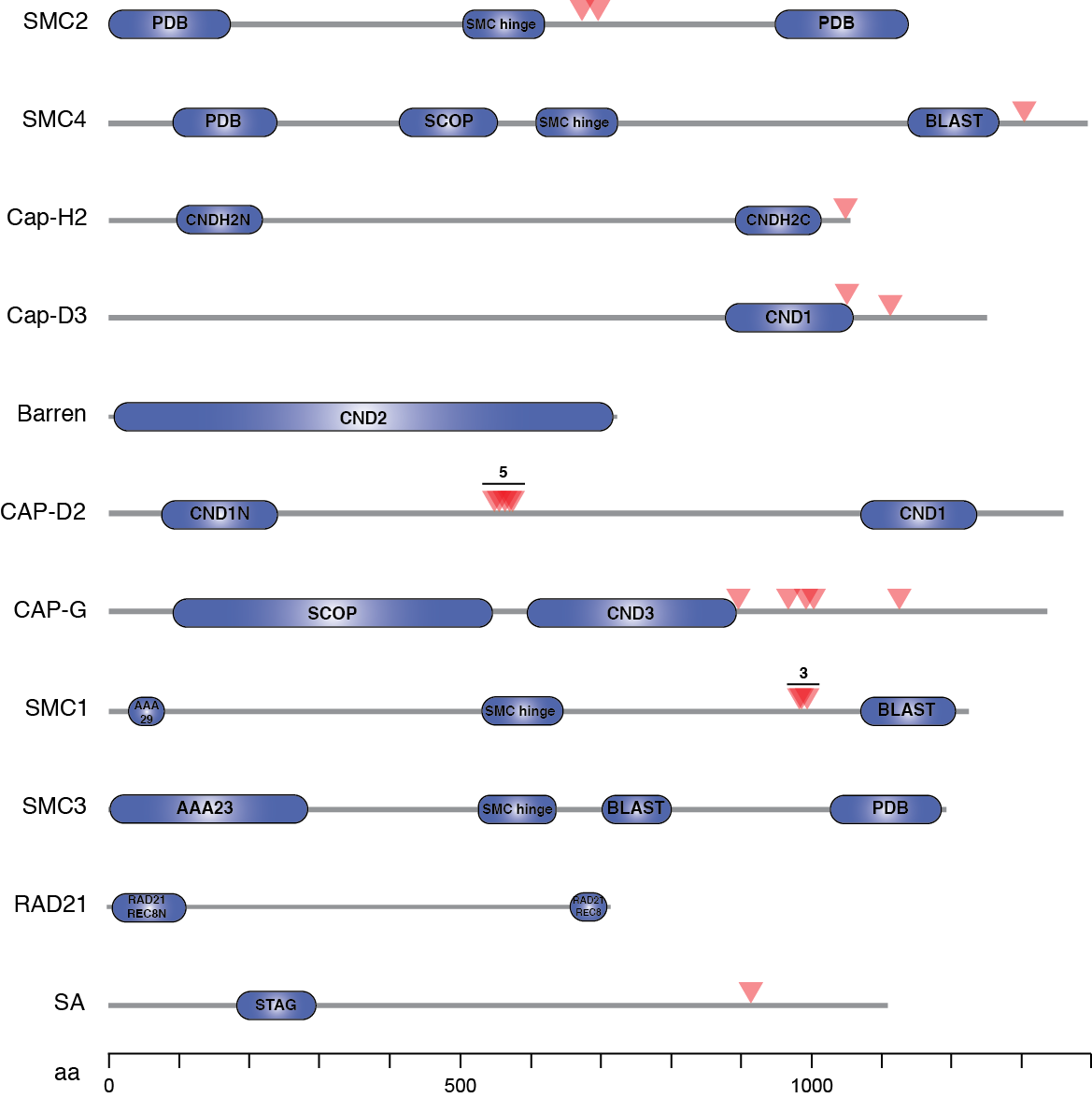


**Figure S7. Sites of repeated positive selection in condensin and cohesin subunits.** Red arrows show residues subject to positive selection (dN/dS) across insect lineages, as indicated by PAML. Boxes indicate putative functional domains as identified by SMART (S10).


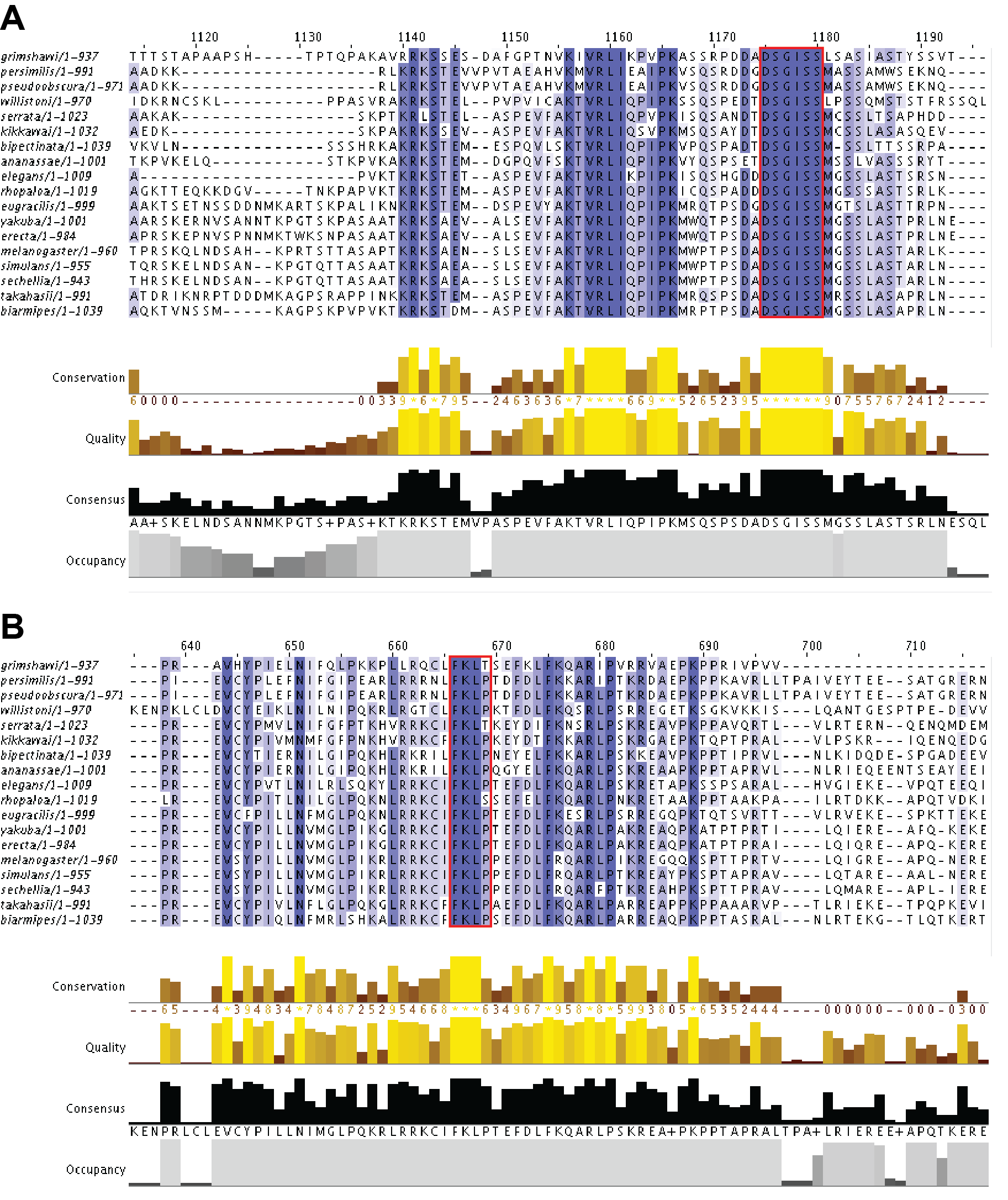


**Figure S8. Cap-H2 regulator binding motifs show strong conservation across *Drosophila*.** Cap-H2 sequences of 18 *Drosophila* species representing ~40 million years of evolutionary divergence were aligned with MUSCLE software and visualized with Jalview (S11, S12). Darker blue represents more highly conserved residues. (A) We found high conservation relative to the surrounding region in the SLMB binding motif (DSGISS, outlined in red). (B) We also observed high conservation in the Mrg15 binding motif (FKLP, in red).


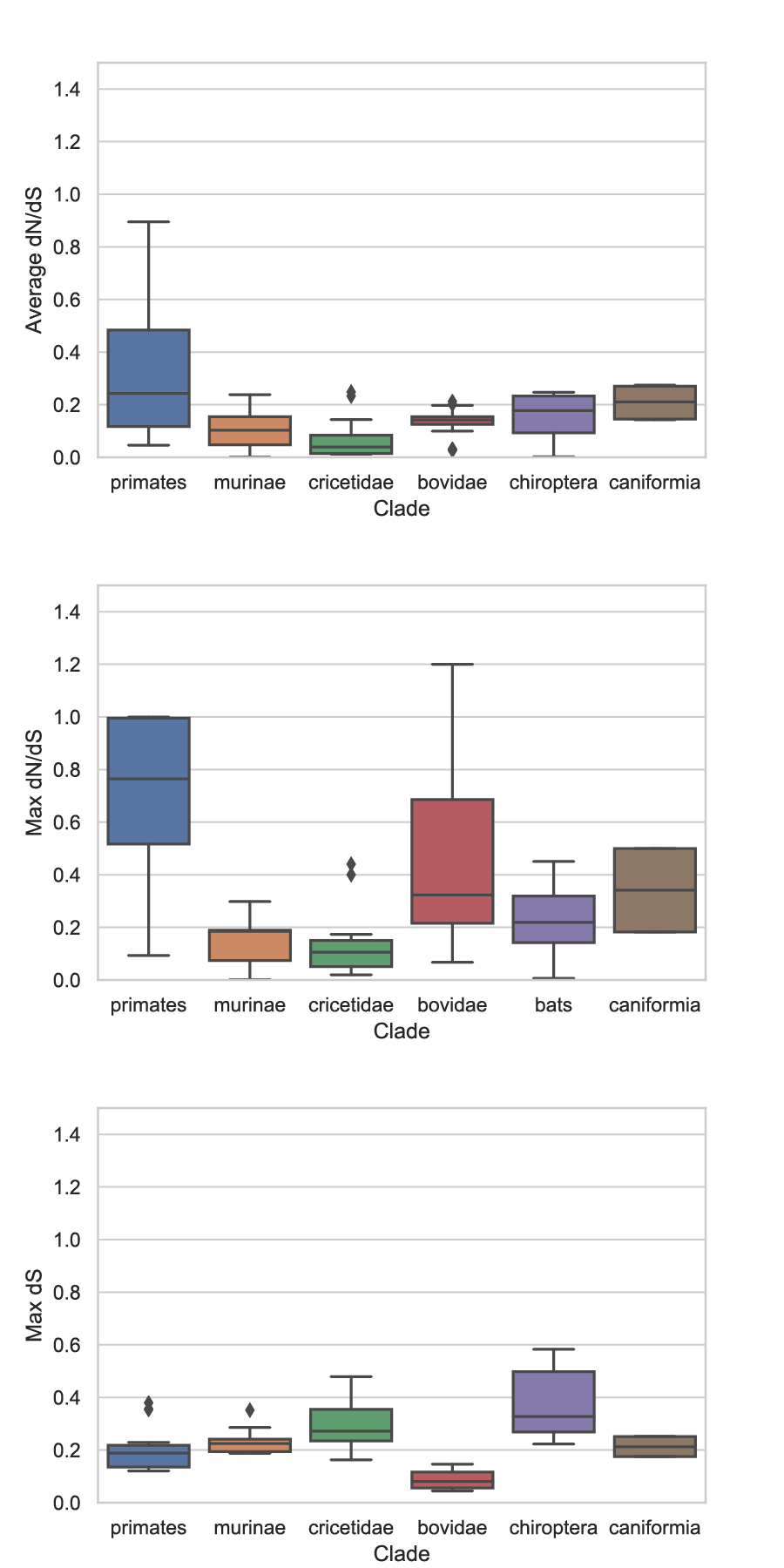


**Figure S9. Amino acid substitution rates for mammal clades used in PAML.** Plots show the distributions of average dN/dS, maximum dN/dS, and maximum dS for all species sampled in each of the mammal clades used in PAML.
